# Care Dynamics of Puerto Rico’s Older Adults: Dementia, Caregiving, Mental Health, and Outmigration

**DOI:** 10.1093/geroni/igaf122.1499

**Published:** 2025-12-31

**Authors:** Michael Crowe, Maricruz Rivera-Hernandez, Michael Crowe

## Abstract

Puerto Rico is experiencing a rapidly aging population due to outmigration and other demographic changes. Older adults in Puerto Rico have a high prevalence of chronic conditions including higher rates of diabetes, heart disease, dementia and depressive symptoms compared to older adults in the US mainland (CDC, 2020). However, compared to the US mainland, Puerto Rico receives lower funding from federal programs including Medicare and Medicaid, which may limit access to quality care, services and support for older adults. This may also impact the health and well-being of caregivers. Understanding the complex relationships between mental health, dementia, caregiving, and social support among older Puerto Rican adults is needed to guide interventions to improve health and well-being of older adults and caregivers. This symposium will feature 5 presentations using various comprehensive datasets including rich demographic and clinical information that provide novel insights and deeper understanding about health and care dynamics faced by older adults in Puerto Rico. Individual presentations will examine: 1) The Prevalence of Alzheimer’s And Related Dementia Among Medicare Advantage Enrollees in Puerto Rico; 2) The Role of Subjective Memory in the Link Between Chronic Pain, Cognitive Decline, and Depressive Symptoms In Older Puerto Rican Adults; 3) Depressive Symptoms in Dementia Caregivers in Puerto Rico: The Role of Caregiver Burden and Symptoms of Dementia; 4) The Association between Social Support and Mental Health-Related Quality of Life among Older Adults in Puerto Rico; and 5) Dementia Diagnosis and Outmigration of Older Puerto Rican Fee-for-Service Beneficiaries.

